# Mine MIMO Depth Receiver: An Intelligent Receiving Model Based on Densely Connected Convolutional Networks

**DOI:** 10.3390/s21248326

**Published:** 2021-12-13

**Authors:** Mingbo Wang, Anyi Wang, Zhaoyang Liu, Heng Zhang, Jing Chai

**Affiliations:** 1 College of Energy Engineering, Xi’an University of Science and Technology, Xi’an 710054, China; lqs_wmb@163.com (M.W.); chaij@xust.edu.cn (J.C.); 2 College of Communication and Information Engineering, Xi’an University of Science and Technology, Xi’an 710054, China; HengZhang1226@163.com

**Keywords:** multiple-input multiple-output system, mine, mine MIMO depth receiver, densely connected convolutional networks

## Abstract

Multiple-input multiple-output (MIMO) systems suffer from high BER in the mining environment. In this paper, the mine MIMO depth receiver model is proposed. The model uses densely connected convolutional networks for feature extraction and constructs multiple binary classifiers to recover the original information. Compared with conventional MIMO receivers, the model has no error accumulation caused by processes such as decoding and demodulation. The experimental results show that the model has better performance than conventional decoding methods under different modulation codes and variations in the number of transmitting terminals. Furthermore, we demonstrate that the model can still achieve effective decoding and recover the original information with some data loss at the receiver.

## 1. Introduction

Coal plays an important role in the production and consumption of primary energy [1]. In recent years, the concept of “intelligent mining” has been introduced to promote the safe and efficient mining of coal. To achieve intelligent coal mining, a complete mobile communication system is essential.

However, unlike surface communication systems, mine tunnels are space-constrained, non-free propagation spaces with complex electromagnetic wave propagation characteristics, which are prone to severe multipath fading [2]. Multipath fading affects the transmission characteristics of signals, causing inter-symbol interference (ISI) and degrading the performance of wireless communication systems [3]. Multiple-input multiple-output (MIMO) communication techniques [4] can effectively counteract the effects of channel fading without increasing the system’s frequency resources and total transmit power, thus reducing ISI.

Some results have been achieved for underground MIMO systems in mines. Liu and his colleagues [5] proposed a MIMO spatially correlated channel model based on Nakagami fading based on the variability of the downhole multipath signal fading characteristics. The Nakagami distribution can be turned into a different distribution by varying the fading index m, which more accurately describes the signal fading characteristics within the mine. In order to improve the communication quality of the MIMO system, Boualleg [6] introduced space-time block coding (STBC) technology to improve the communication quality. The pilot frequency uses the minimum mean square error (MMSE) principle to estimate the underground MIMO channel. The resulting MIMO channel estimate is used to evaluate the data portion of the STBC block, and a maximum likelihood (ML) detector is used to perform the decision. In addition, Boualleg used a Monte Carlo simulation to study the reasons for the degradation of system BER performance due to MMSE estimation errors. Moreover, Yang and his colleagues [7,8] researched and improved the channel coding method to improve the performance of the MIMO system.

In recent years, with the development of artificial intelligence, the application of deep learning models to communication systems has become a current research hotspot [9,10]. The literature [11,12,13] proposes a new channel estimation method that treats the channel matrix as a two-dimensional image and achieves channel estimation by image processing through neural networks. The literature [14] applies neural networks to the study of modulation recognition, and achieves channel modulation recognition by convolutional neural networks. The above research mainly uses deep learning or other algorithms to optimize and replace a certain process in the traditional communication method. In order to improve the performance of the underground MIMO system, this paper designs an end-to-end mine MIMO system deep receiver model, and our contributions are as follows:

- We propose a new mine MIMO depth receiver model, which is based on densely connected convolutional networks for data feature extraction, while multiple binary classifiers are constructed to achieve end-to-end data recovery. To achieve end-to-end signal recovery, the model takes as input the IQ signal received by the receiver of the MIMO system, and the output data consist of the original bit stream from the transmitter. We believe that when this model is heavily trained, it has a low BER. Moreover, the model is suitable for the blind reception of multiple modulation and coding methods, and offers superior performance compared to conventional MIMO receivers.
- The mine MIMO depth receiver, which uses the same modulation coding method in different mine environments, achieves and surpasses the traditional decoding method. First, we build the mine MIMO communication system to generate training data and test data, and then the IQ signal received by the receiver of the MIMO system is used as the input of the mine depth receiver model, and the raw bitstream data of the transmitter are used as the output for network training. The results show that the mine MIMO depth receiver has a higher decoding performance compared to the traditional decoding method. In addition, the mine MIMO depth receiver is not affected by the channel environment, and the receiver maintains a low BER under different channel environments.
- The scheme proposed in this paper allows for high quality decoding in the same mine environment by using different modulation coding methods. Firstly, we build datasets with different decoding methods in the same mine environment, and then train the model. The results show that the scheme can achieve decoding effectively for different modulation coding schemes, while its decoding performance is better than that of traditional decoding schemes.
- When the number of antennas at the transmitting end of the MIMO communication system changes, the mine MIMO depth receiver model is not affected by the number of antennas. At the same time, the model has a higher decoding performance than the traditional method. Firstly, the datasets under the transmitting antennas of 2, 3, and 4 are built. Then the model is trained. The results show that the mine MIMO depth receiver has a higher decoding performance relative to the traditional decoding method, and the change of the number of transmitters has less effect on the performance of the mine MIMO depth receiver.
- When there are missing data at the receiver end, the mine MIMO depth receiver can still achieve accurate decoding under certain conditions. First, we build different missing datasets, and then train and validate the network. The results show that the mine MIMO depth receiver can still achieve accurate decoding despite missing data. In other words, a communication system with a mine MIMO depth receiver can reasonably reduce the data at the transmitter side while maintaining the decoding performance of the system, which will offer the possibility of reducing the power consumption of the MIMO system.

The rest of the paper is organized as follows. Section 2 presents background information on the MIMO communication system and the mine communication channel used and constructed in this paper. Section 3 explains, in detail, the process of building the mine MIMO depth receiver model. The experimental results of the model under different conditions are presented in Section 4, and the conclusions are given in Section 5.

## 2. Theoretical Basis

This section briefly describes the basic principles of MIMO communication technology and the construction of the mine MIMO channel model. These technologies are the basis for subsequent research.

### 2.1. MIMO Signal Model

**Figure 1:**
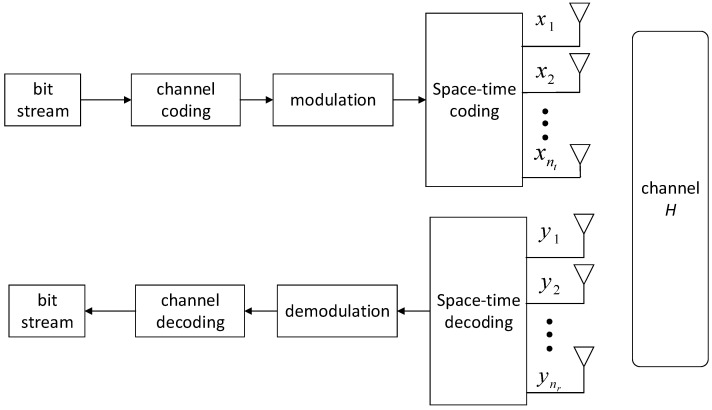
Schematic diagram of the MIMO system.

In the process of sending information at the MIMO transmitter, the information bitstream processed and converted by voice, text, image, etc., is first channel-coded, modulated, and pulse-shaped, and then after space-time coding, different antennas radiate the generated signals into the air. However, the receiving end processes the data in the opposite way to the sending end. After the receiver receives the signal, it uses demodulation, channel decoding, and other methods to finally recover the information code stream. The schematic diagram of the MIMO depth receiver is shown in Figure 1, below. Moreover, for a communication system that adopts adaptive coding and modulation at the transmitting end, the receiving end often needs to know which modulation and coding method the current signal adopts in order to select the corresponding receiving algorithm for information recovery. Assuming that the transmitter has $n_{t}$ antennas and the receiver has $n_{r}$ antennas, the input and output relationship of the MIMO channel [15] is shown in the following Formula (1):

(1) y=H·x+n

where $x=[x_{1},x_{2}\cdots x_{n_{t}}]$ is the signal transmission vector of $1\times n_{t}$, *n* is Gaussian additive white noise, and *y* is the received information vector. In the above formula, *H* is represented as an $n_{t}\times n_{r}$ matrix, which represents the channel gain of each transmitting and receiving antenna pair, and *H* is represented as shown below:

(2) $$H=\left[\begin{array}{l} h_{1,1}\cdots h_{1,n_{r}}\\ \;\vdots \;\;\;\vdots \\ h_{n_{t},1}\cdots h_{n_{t},n_{r}} \end{array}\right]$$

### 2.2. Space-Time Coding Technology

In the MIMO system, there are many space-time coding schemes. In this paper, we will introduce the space-time block code scheme to obtain the best reliability. In this research, the most representative space-time block code, Alamouti, is selected. The principle of the coding scheme is as follows. Taking dual antennas at the transmitting end as an example, for the symbol group $s=[s_{1},s_{2}]$ that needs to be transmitted at a certain moment: in the first time slot, $s_{1}$ is transmitted through the first antenna, and $s_{2}$ is transmitted through the second antenna. In the next time slot, $-s_{2}^{*}$ is transmitted from the first antenna, and $s_{1}^{*}$ is transmitted from the second antenna. Therefore, the data sent by each antenna in each time slot can be expressed by the following matrix [16]:

(3) $$X=\left[\begin{array}{l} s_{1},-s_{2}^{*}\\ s_{2},s_{1}^{*} \end{array}\right]$$

In the above formula, each column of the matrix represents the data sent by each antenna in each time slot.

When the receiving end is multi-antenna, assuming that in the *k*-th time slot, the data received by the *j*-th antenna is $y_{j}(k)$, and the channel factor from the *i*-th transmitting antenna to the *j*-th receiving antenna is expressed as $h_{i,j}$, then the signal received by the *j*-th antenna in the first time slot is as follows:

(4) y(1)j=(h1,js1+h2,js2)+nj(1)

The signal received in the second time slot is as follows:

(5) y(2)j=(−h1,js2*+h2,js1*)+nj(2)

In the above formula, $n_{j}(1)$ and $n_{j}(2)$ represent the additive white Gaussian noise samples in the first and second time slot channels, respectively.

### 2.3. The Channel Model

There is a phenomenon of multipath fading in the signal transmission in the mine. Different from the ground environment, the propagation of electromagnetic waves in the mine is easily affected by the inclination angle of the roadway wall, the roughness of the roadway wall, the dust droplets in the roadway, and the supporting materials [17,18]. The signal sent by the transmitter arrives at the receiving end after scattering, reflection, and diffraction. The time, amplitude, and phase of each signal reaching the receiver are also different. The interaction between the signals causes the instantaneous received signal amplitude and phase. Random fluctuations produce multipath fading.

Mine tunnels can generally be divided into long straight tunnels and curved tunnels. When the transmitting and receiving antennas are located in a long and straight roadway, the multipath signal fading approximately obeys the Rician distribution; when the transmitting and receiving antennas are located in the main roadway and the branch roadway, the multipath signal fading approximately obeys the Rayleigh distribution [5]. In this paper, the Nakagami channel with variable parameters is used as the mine channel model. In Formula (6), the channel gain of any antenna is shown in the following formula:

(6) $$H_{i,j}=h_{i,j}\times \exp(j\varphi_{i,j})$$

In the above formula, $1\leq i\leq n_{t}$, $1\leq j\leq n_{r}$, $h_{i,j}$ represents the amplitude of the channel gain, and $\varphi_{i.j}$ means phase. The average power of the normalized channel gain amplitude is $E(h_{i,j}^{2})=1$. If the probability density function of $h_{i.j}$ is Formula (7), then the channel is said to obey the Nakagami distribution:

(7) $$f_{R}(a)=\frac{2}{\Gamma (m)}{\left(\frac{m}{\Omega }\right)}^{m}a^{2m-1}\exp(-\frac{ma^{2}}{\Omega })$$

Γ(m) is the Gamma function, m is the Nakagami parameter, and m≥0.5, $\Omega =E(a^{2})$ represents the instantaneous power.

## 3. Mine MIMO Depth Receiver

**Figure 2:**
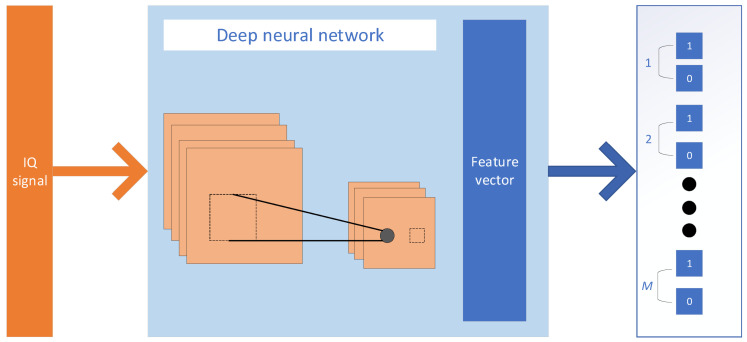
Schematic diagram of the mine MIMO depth receiver model.

This section will introduce the mine MIMO depth receiver, which is designed with reference to the deep receiver model proposed in the literature [19], and a schematic diagram of the model is shown in Figure 2. For the original bitstream with M bits at the transmitter, the IQ signal received at the receiver end is first feature extracted, and then this feature vector is input to M binary categories where the recovery of the original bitstream is finally achieved.

### 3.1. Densely Connected Convolutional Networks

**Figure 3:**
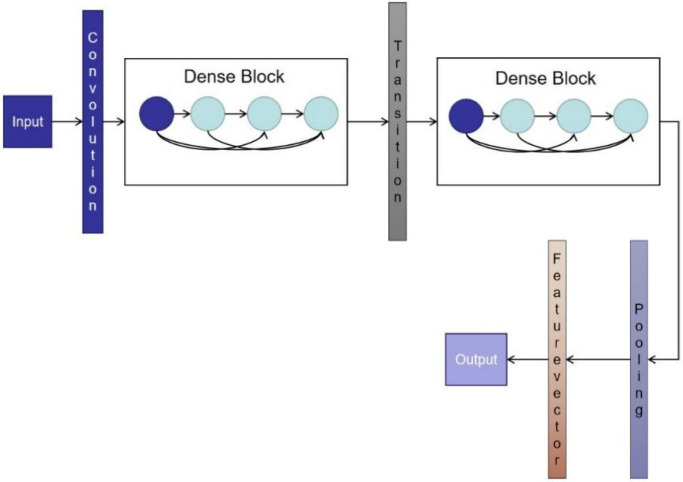
Basic structure of DenseNet.

The mine MIMO depth receiver is built by means of a densely connected convolutional network (DenseNet). DenseNet was proposed by Huang and his colleagues [20]. The network is a unique convolutional neural network where each layer in the network is connected to other layers, and each layer of the network receives the output information from all previous layers. The features extracted from this layer are passed to the subsequent layers, ensuring that the maximum information flow is obtained between the layers. DenseNet has the advantage of feature reuse and enhanced transfer of feature values compared to other convolutional neural network (CNN) structures, and in addition, DenseNet can effectively mitigate the problem of gradient disappearance. The DenseNet network is shown in Figure 3. The DenseNet network is mainly composed of dense blocks and transition layers.

**Figure 4:**
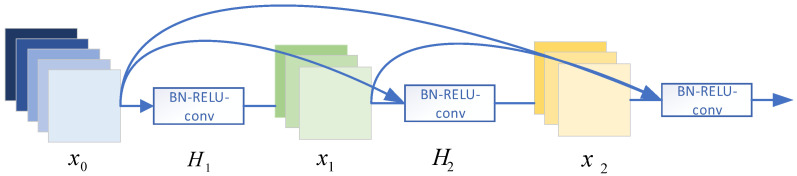
Three-layer dense block model.

Dense blocks: Figure 4 shows the three-layer dense block model. Compared with the traditional l-layer network model, the dense link network has $\frac{l(l-1)}{2}$-layer connections. Moreover, the input of each layer is related to all of the network outputs of the previous layer, which will effectively avoid the problem of the gradient disappearing with the increase of network layers. For DenseNet, its layer l output is as follows:

(8) $$X_{l}=H_{l}\left[x_{0},x_{1},\cdots ,x_{l-1}\right]$$

$X_{l}$ represents the output feature of layer l, and $\left[x_{0},x_{1}\cdots x_{l-1}\right]$ represents the cascade of features acquired from layer 0 to Layer l−1 networks.

Transition layer: The layer between adjacent dense blocks is called the transition layer. Due to the different characteristics of the output of each dense block, the transition layer is set to ensure the unity of the characteristic dimensions of the output of the dense block. It can be seen from Figure 4 that the transition layer is mainly composed of normalization, RELU, and convolutional and pooling layers.

### 3.2. Mine MIMO Depth Receiver Model

**Figure 5:**
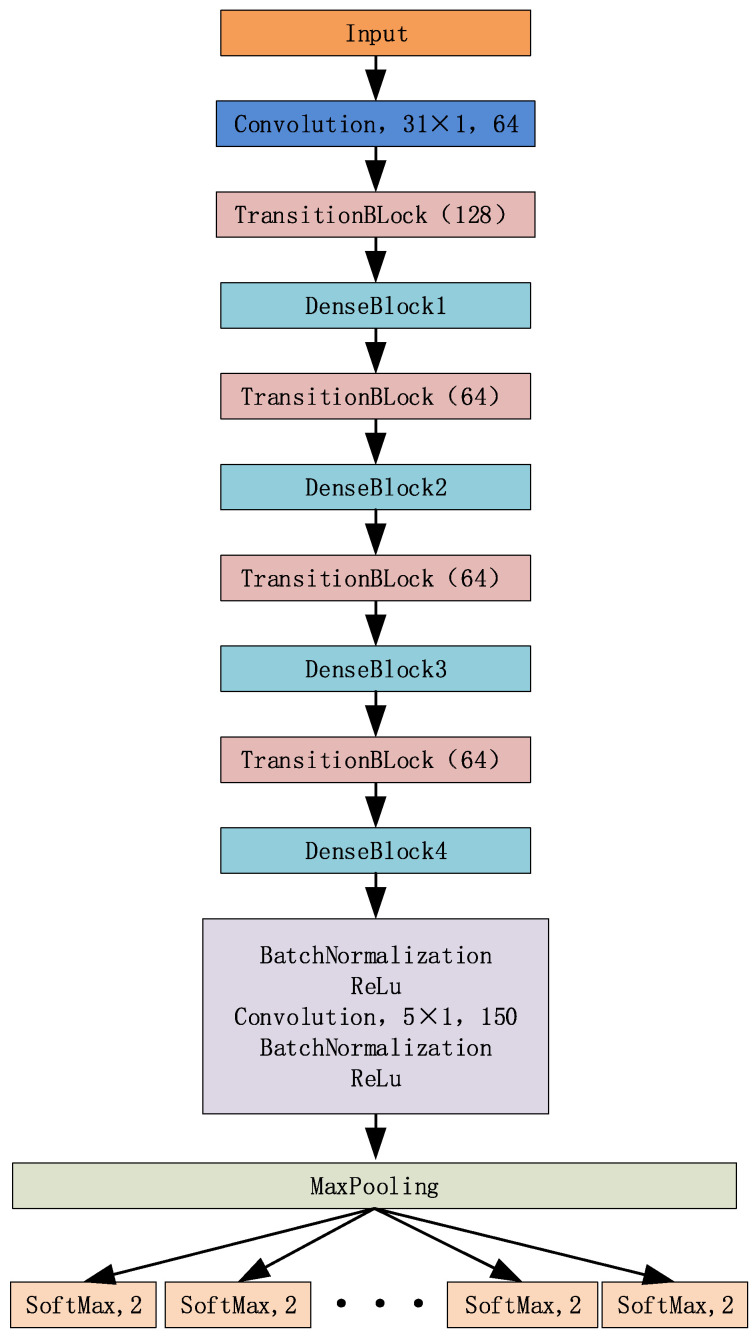
Mine MIMO depth receiver.

Inspired by the literature [19], a mine MIMO depth receiver (MMDR) model is designed, which uses a dense neural network for IQ data stream information feature extraction, and M binary classifiers for the extracted feature vectors to achieve the recovery of the original data bits. In addition, unlike the 2D CNN network structure used in image classification, the MMDR model uses a 1D CNN structure for feature extraction, which has the advantage that the IQ data stream can be directly recovered from the original data bits without pre-processing. The MMDR model is shown in Figure 5.

Figure 5 is the MMDR model built in this paper, which contains four dense blocks and four TransitionBlock(K), where K represents the size of the convolution kernel in TransitionBlock. For the four dense connection blocks in the figure, their structures are shown in Table 1. Moreover, in DenseBlock and TransitionBlock, the size of the convolution kernel is 5 × 1.

After the data feature extraction is completed in the densely connected network, the data are input into the fully connected network. Then, for the M-bit data, M binary classifiers are set up to realize the signal recovery. Since M binary classifiers are used to realize bitstream recovery, the problem of too many categories can be solved by using a single classifier with too many bits of information. Assuming that the length of the original bitstream data is 16, the number of labels required by adopting a single classifier is 2^16^ = 65,536. It is difficult to design and train a neural network, mainly reflected in two aspects. Firstly, the number of hidden nodes at the last classification layer of the neural network is generally consistent with the number of categories; including such a large number of hidden nodes increases the complexity of the network’s space and time. Secondly, for each category, certain training samples are often required. Therefore, the number of training samples is far greater than 2^M^ and thus it is uneconomical to generate such a large number of training samples. Moreover, the computational complexity of the training will increase significantly, and the network will be difficult to converge in a limited time [11].

## 4. Model Performance Analysis

In the MMDR model validation session, the mine MIMO communication system model was first built in the MATLAB R2020 environment, and then the MMDR model was built in the Keras framework using the Python language.

To verify the decoding performance of the MMDR in different mine environments, different fading coefficients m under the Nakagami channel were selected, while the transmitter original information bit length was set to 32 and randomly generated. The channel coding method was (7, 4) Hamming channel coding with BPSK modulation, and the number of transmitter–receiver antennas was set to 2. The signal Eb/N0 range was from 0 dB to 8 dB with a step of 1 dB, and the number of Eb/N0 samples for each of the training and test sets was 80,000. *m* = 0.5, *m* = 1, and *m* = 2 for the different fading coefficients in the Nakagami channel, indicating different channel environments: *m* = 0.5 for one-sided Gaussian distribution, *m* = 1 for Rayleigh distribution, which indicates the amplitude fading characteristics of the multipath signal amplitude in curved lanes, and *m* = 2, indicating the amplitude fading characteristics of the multipath signal in long straight lanes.

**Figure 6:**
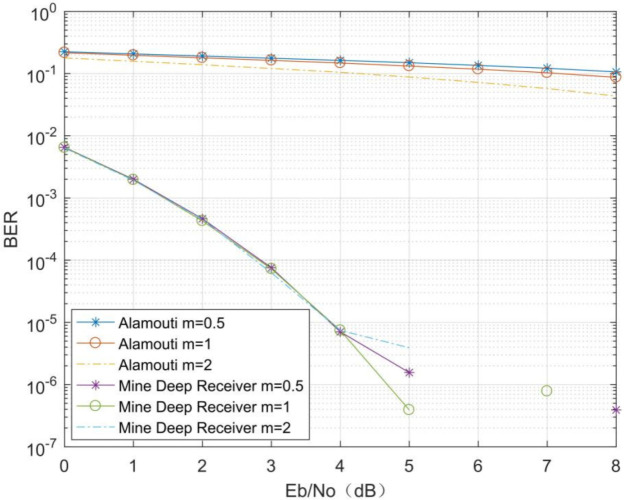
Comparison of the decoding performance in different channel environments.

The experimental results (Figure 6) show that the MMDR model has better decoding performance compared to the traditional Alamouti decoding method in different channel environments. The traditional Alamouti decoding method, which is affected by the channel environment, cannot achieve accurate decoding, and the Alamouti decoding accuracy gradually improves as the Nakagami channel fading factor m increases. However, the mine MIMO depth receiver proposed in this paper is designed with an end-to-end idea, and there is no error accumulation in its decoding process, so it is better than the Alamouti decoding method. For different channel environments, the BER of the MMDR model is close to 10^−2^ for a bit signal-to-noise ratio (Eb/N0) 0, which is more resistant to interference. At higher Eb/N0s, the BER of the MMDR model is approximately 0, which is significantly better than the traditional Alamouti decoding method.

**Figure 7:**
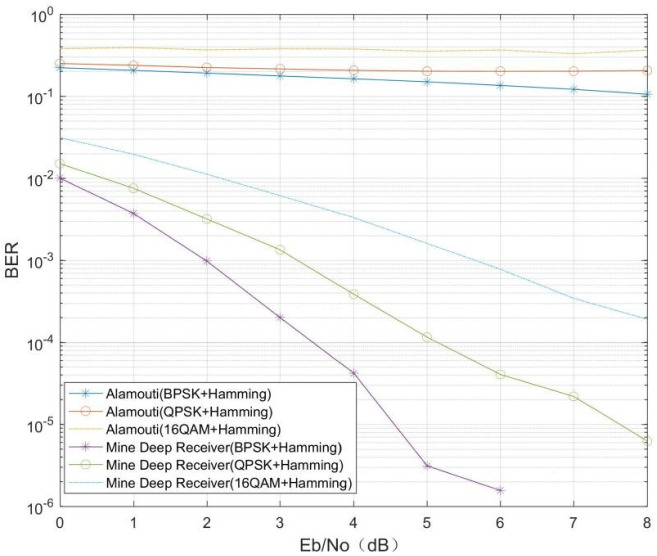
(7, 4) Hamming channel coding.

**Figure 8:**
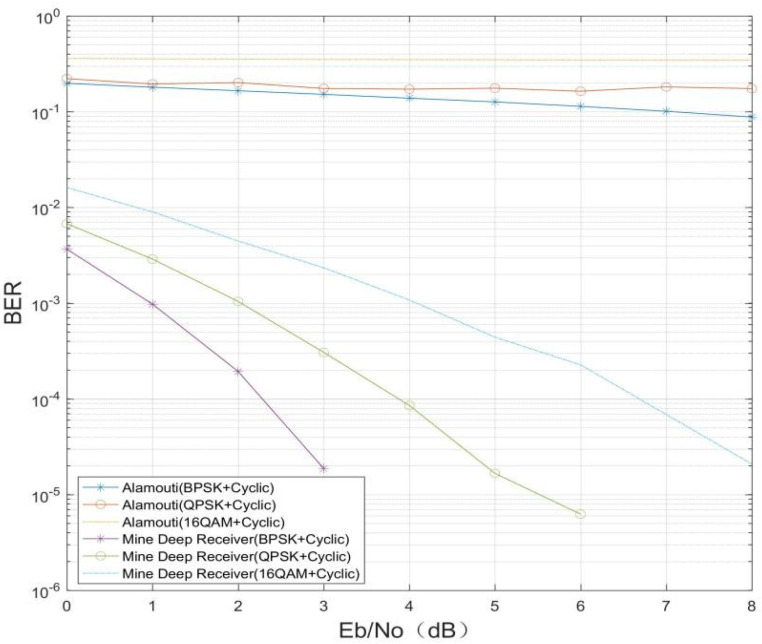
(7, 3) Cyclic channel coding.

The effect of different modulation and coding methods on the decoding capability of the MMDR model was verified. The fading factor *m* = 0.5 under the Nakagami channel, the length of the original message bit data was 32, and the modulation and coding methods are shown in Table 2, below. The BPSK+(7, 4) Hamming code, QPSK+(7, 4) Hamming code, 16QAM+(7, 4) Hamming code, BPSK+(7, 3) cyclic code, QPSK+(7, 3) cyclic code, and 16QAM+(7, 3) cyclic code were selected. The number of transmitter–receiver antennas was set to 2, and the signal Eb/N0 range was from 0 dB to 8 dB in 1 dB steps, with 20,000 samples per Eb/N0. The experimental results are shown in Figure 7 and Figure 8, below.

Figure 7 and Figure 8 show that the conventional Alamouti decoding method is less effective in the same channel environment and with different modulation methods, in addition to the fact that the conventional Alamouti decoding is affected by the channel coding as well as the modulation method. However, for the MMDR model, recovery of the original bits of information can be effectively achieved, and its performance is better than that of conventional receivers for the same modulation and channel coding. In addition, comparing Figure 7 and Figure 8, there is some difference in the BER of the MDMR model under different modulation coding. The reason for this phenomenon is that for the same length of binary raw bit data, with different channel coding and modulation, the data length received at the receiver end is different, and for the MMDR model, there are differences in the data features extracted from the IQ data stream, so the mine MIMO depth receiver is affected to some extent by the channel coding and modulation method.

To verify the effect of the number of transmitting antennas on the MIMO depth receiver at the mine, the original information bit data length at the transmitter was set to 24, the channel coding method was (7, 4) Hamming channel coding, and the modulation method was BPSK modulation. The signal Eb/N0 ranged from 0 dB to 8 dB in steps of 1 dB, with 80,000 samples per Eb/N0. The Nakagami channel fading factor was *m* = 0.5, the number of transmitter antennas was set to 2, 3, and 4, and the number of receiver antennas was set to 2.

**Figure 9:**
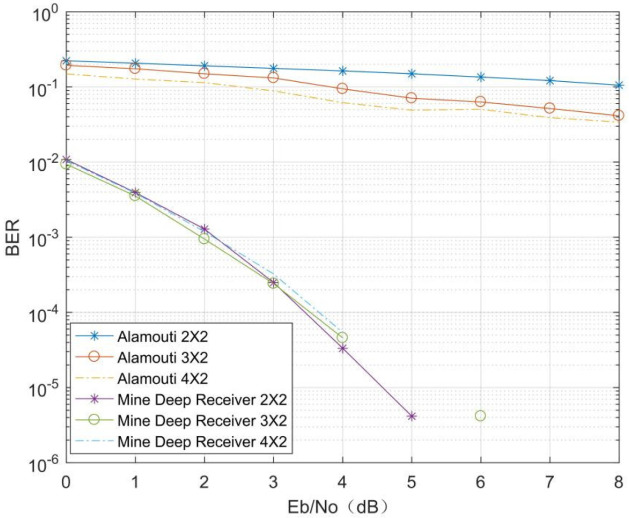
Comparison of different transmitting antennas.

As can be seen from Figure 9, the conventional Alamouti decoding BER decreases as the number of transmitting antennas increases. For the MMDR model, the decoding accuracy is significantly due to the conventional decoding method. When the Eb/N0 is low, the BER of the MMDR model is much higher than that of the traditional Alamouti decoding method, and when the Eb/N0 reaches 6 dB or more, the BER of the MMDR model is zero. In addition, for the mine MIMO depth receivers, the decoding accuracy curve shifts slightly and stays within the same magnitude when the number of transmitter antennas increases. For the MIMO communication systems, the MMDR model can maintain a low BER and achieve accurate decoding with a small number of antennas at the transmitter.

**Figure 10:**
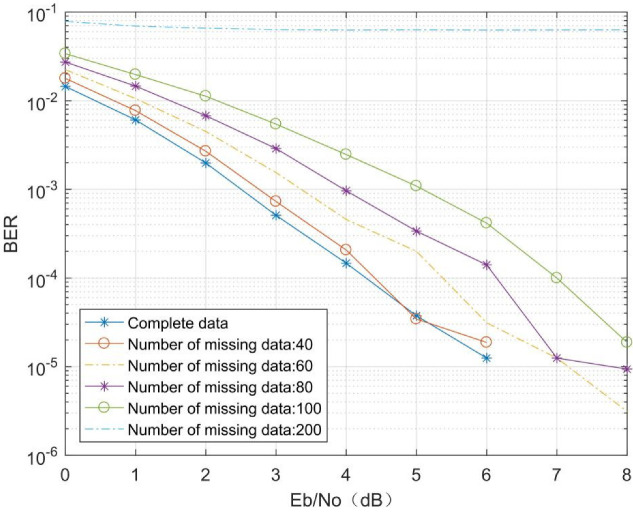
Decoding comparison of different missing data depth receiver.

To verify the effect of missing data at the transmitter on the MMDR model, a channel environment with a fading factor of *m* = 0.5 under the Nakagami channel was selected, with 32 bits of raw message bit data, a pulse-forming filter roll-off factor of 0.5, 8 sampling points per symbol, via the BPSK+(7, 4) Hamming code, and the number of transmitter–receiver antennas both set to 2. The MIMO receiver received an IQ signal length of 448 per antenna, which gives a model input data length of 1792 for the MMDR model. The signal Eb/N0 ranged from 0 dB to 8 dB in steps of 1 dB, and the number of samples per Eb/N0 was 10,000. The missing data were set to 40, 60, 80, 100, and 200. The experimental results are shown in Figure 10, below.

As can be seen from Figure 10, the MMDR model has a BER that approximates the decoding BER with complete data when the missing data at the receiver side is 40. As the missing data at the receiver side gradually increases, the MMDR model BER increases significantly when the missing data reach 100, but is still lower than the Alamouti BER in the same environment shown in Figure 6. When the missing data at the receiver side are 200, the MMDR model fails to achieve signal decoding. The above experiments show that the MMDR model proposed in this paper can accurately decode the signal under the condition that the received signal is missing, which provides the possibility to reduce the power consumption of the MIMO system while achieving low BER.

## 5. Conclusions

In this paper, a mine MIMO depth receiver model is proposed. This model replaces the overall recovery process of the traditional MIMO receiver from the IQ signal to the information bits. Compared with the traditional MIMO receiver, this model has the following characteristics:

- In different mine environments, the mine MIMO depth receiver has stronger decoding performance. The simulation results show that the depth receiver has higher decoding performance in three different mine environments, and its decoding performance is not affected by the environment. In other words, the mine MIMO depth receiver has stronger anti-interference performance.
- Under different channel coding and modulation, the mine MIMO depth receiver has stronger decoding performance.
- The decoding performance of the mine MIMO depth receiver is not affected by the number of antennas at the transmitting end. The simulation results show that as the number of transmitter antennas increases, the decoding performance of the traditional MIMO receiver is improved, while the deep receiver model is not affected by the number of transmitter antennas, and its decoding performance remains at the same order of magnitude.
- The mine MIMO depth receiver can restore the original information when the data at the receiving end is lost. In other words, the communication system using the mine MIMO depth receiver can cut the transmitting end data reasonably, which will provide the possibility to reduce the power consumption of the MIMO system.

## Figures and Tables

**Table 1:** DenseBlock structure.

DenseBlock(K)	Number of Network Layers	Number of Convolution Kernels
DenseBlock1	2	128
DenseBlock2	3	64
DenseBlock3	4	64
DenseBlock4	3	64

**Table 2:** Sample of different modulation coding methods.

Dataset Category	Modulation Method	Channel Coding Method	Number of Samples
1	BPSK	(7, 4) Hamming code	20,000 × 9
2	QPSK	(7, 4) Hamming code	20,000 × 9
3	16QAM	(7, 4) Hamming code	20,000 × 9
4	BPSK	(7, 3) cyclic code	20,000 × 9
5	QPSK	(7, 3) cyclic code	20,000 × 9
6	16QAM	(7, 3) cyclic code	20,000 × 9

## Data Availability

Not applicable.
